# The complete chloroplast genome of *Turpinia arguta* (Staphyleaceae)

**DOI:** 10.1080/23802359.2020.1841579

**Published:** 2020-12-27

**Authors:** Lan Cao, Zejing Mu, Xianping Yang, Zhongqing Guo, Xiaolang Du, Fang Liang

**Affiliations:** a Research Center for Traditional Chinese Medicine Resources and Ethnic Minority Medicine, Jiangxi University of Traditional Chinese Medicine, Nanchang, China; b YuZhang Normal University, Nanchang, China

**Keywords:** Complete chloroplast genome, *Turpinia arguta*, Staphyleaceae

## Abstract

The complete chloroplast genome of *Turpinia arguta* was sequenced and assembled for the first time. The chloroplast genome was 160,139 bp in length, containing a large single-copy region (LSC) of 89,625 bp and a small single-copy region (SSC) of 18,262 bp, separated by a pair of inverted repeats (IRs) of 26,126 bp. The genome contained 113 unique genes, including 79 protein-coding genes, 30 tRNA genes, and four rRNA genes. Among them, 15 genes had one intron each and 3 genes containing two introns. The overall GC content was 37.4%, while the corresponding values of LSC, SSC, and IR regions were 35.4%, 31.8%, and 42.8%, respectively. Phylogenetic analysis showed that *T. arguta* is more closely related to *Staphylea trifolia* and provided new insight into the evolution of Staphyleaceae.

*Turpinia* Ventenat is the largest genus of Staphyleaceae (Li et al. 2008), with 30–40 species in the world. There are 13 species of *Turpinia* in China, among which *Turpinia argute* Seem., has a long history on clinical applications. *Turpinia arguta* is often used in the treatment of tonsillitis and pharyngitis (Xiao et al. 2019). In this study, we sequenced and assembled the chloroplast genome of *T. arguta* for the first time, which is also the first complete chloroplast genome in the genus *Turpinia*.

Fresh leaves of *T. arguta* were collected from Yongxin, Jiangxi, China (GPS: 26°53′10.261″, 114°17′25.811″). Herbarium voucher (Voucher No. JZ2020050101) was deposited in Medicinal Herbarium, Jiangxi University of Traditional Chinese Medicine, Nanchang, China. Total genomic DNA was extracted using the modified CTAB method (Doyle and Doyle 1987) and sequenced on an Illumina NovaSeq platform with paired-end reads of 150 bp. A total of 33,923,324 clean reads (5 Gb) were obtained. The sequencing quality evaluation showed that the percentage of bases with quality value ≥30 (Q30) was 92.5%. The GetOrganelle pipeline (Jin et al. 2020) was carried out for the de novo assembly of chloroplast genome. Genes were annotated by PGA (Qu et al. 2019) and visually checked in Geneious v8.0.2 (Kearse et al. 2012) using chloroplast genome of *Staphylea trifolia* (GenBank accession MK488092) as reference. The predicted transfer RNAs (tRNAs) were confirmed by tRNAscan-SE 2.0 (Lowe and Chan 2016). Finally, the complete chloroplast genome with annotations was submitted to GenBank (accession MT859133). Raw reads were deposited in the GenBank Sequence Read Archive (SRA SRR12717619).

The size of complete chloroplast genome of *T. arguta* is 160,139 bp with high coverage (mean 912×). It has a typical quadripartite structure, including a large single-copy region (LSC) of 89,625 bp, a small single-copy region (SSC) of 18,262 bp, and a pair of inverted repeats (IRs) of 26,126 bp. There are 79 protein-coding genes, 30 tRNA genes, and four rRNA genes. Among these genes, 15 of them (*atpF*, *ndhA*, *ndhB*, *petB*, *petD*, *rpl2*, *rpl16*, *rpoC1*, *rps16*, *trnA-UGC*, *trnG-UCC*, *trnI-GAU*, *trnK-UUU*, *trnL-UAA* and *trnV-UAC*) are single-intron genes, and three genes (*clpP*, *rps12* and *ycf3*) contain two introns. The overall GC content is 37.4%, while the GC content of LSC, SSC, and IR regions are 35.4%, 31.8%, and 42.8%, respectively.

To identify the phylogenetic relationship of *T. arguta* in malvids, the phylogenetic tree including three species of Crossosomatales, and 13 species of seven other orders within malvids were reconstructed using complete chloroplast genomes. The sequences were aligned by MAFFT v7.017 plugin (Katoh et al. 2002) and visually checked in Geneious. Phylogenetic analysis was performed by RAxML v8.2 (Stamatakis 2014) using 1000 replicates of a rapid bootstrap analysis with GTRGAMMA substitution model. The phylogenetic relationships among all orders of malvids were fully resolved with maximum support (Figure 1). Among them, *T. arguta* is more closely related to *Staphylea trifolia* and then grouped with *Euscaphis japonica.* The chloroplast genome obtained in this study could provide essential data to the evolution of Staphyleaceae.

**Figure 1.**
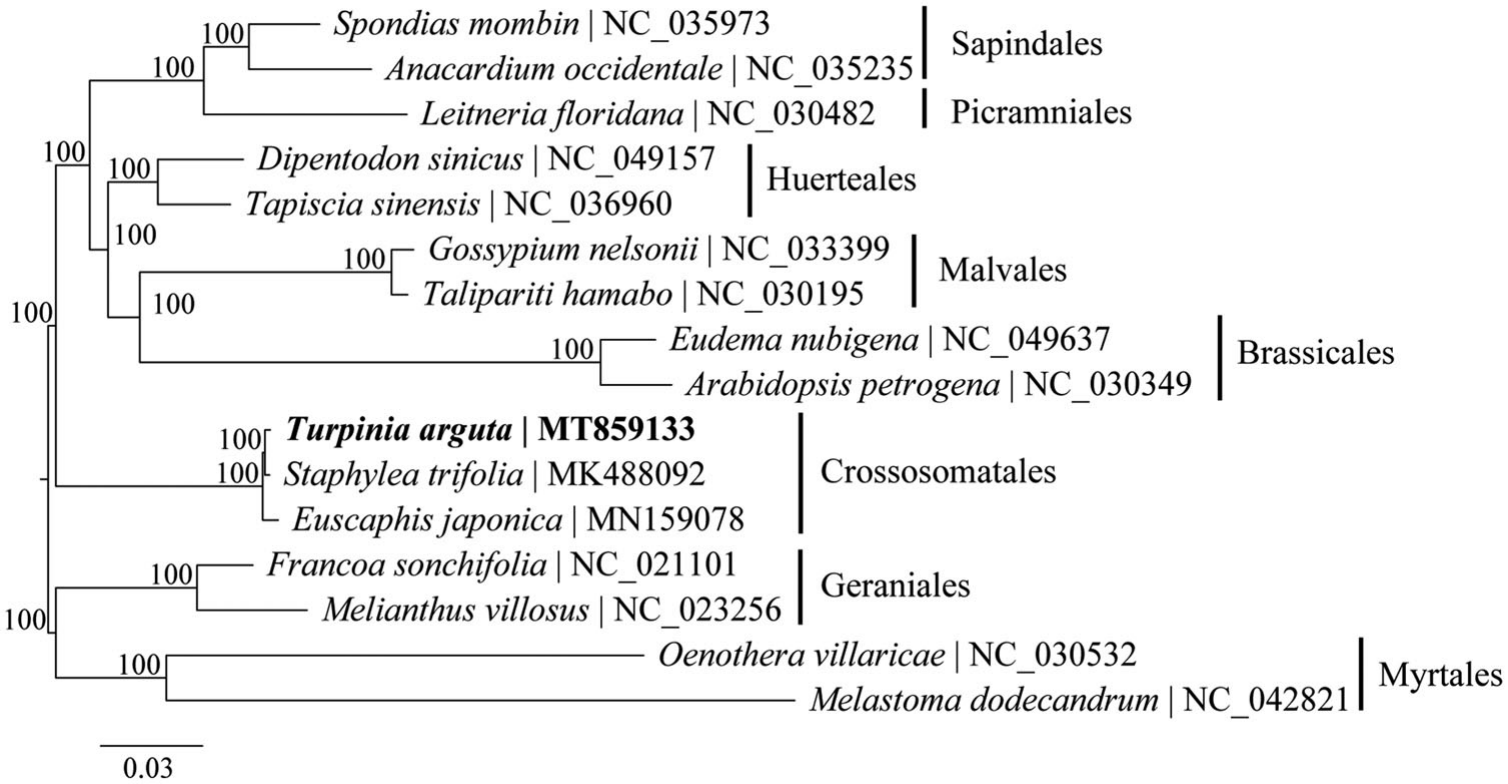
Maximum-likelihood phylogenetic tree based on complete chloroplast genomes. Numbers close to each node are bootstrap support values.

## Data Availability

The data that support the findings of this study are openly available in GenBank at https://www.ncbi.nlm.nih.gov/genbank/, accession numbers [MN159078, NC_030349, NC_049637, NC_035235, NC_048982, NC_042821, NC_030532, NC_021101, NC_023256, MK488092, NC_036960, NC_049157, NC_030482, NC_030195, NC_033399 and NC_035973]. The complete chloroplast genome generated for this study has been deposited in GenBank with accession number MT859133. All high-throughput sequencing data files are available from the GenBank Sequence Read Archive (SRA) accession number: SRR12717619.
